# Silver Nanoparticles: Two-Faced Neuronal Differentiation-Inducing Material in Neuroblastoma (SH-SY5Y) Cells

**DOI:** 10.3390/ijms19051470

**Published:** 2018-05-15

**Authors:** Ahmed Abdal Dayem, Soo Bin Lee, Hye Yeon Choi, Ssang-Goo Cho

**Affiliations:** Department of Stem Cell and Regenerative Biotechnology, Incurable Disease Animal Model & Stem Cell Institute (IDASI), Konkuk University, Seoul 05029, Korea; ahmed_morsy86@yahoo.com (A.A.D.); soobineey@naver.com (S.B.L.); hyeon.choi24@gmail.com (H.Y.C.)

**Keywords:** silver nanoparticles, neuroblastoma, differentiation, kinase, phosphatase, reactive oxygen species, mitochondria

## Abstract

We have previously demonstrated the potential of biologically synthesized silver nanoparticles (AgNP) in the induction of neuronal differentiation of human neuroblastoma, SH-SY5Y cells; we aimed herein to unveil its molecular mechanism in comparison to the well-known neuronal differentiation-inducing agent, all-trans-retinoic acid (RA). AgNP-treated SH-SY5Y cells showed significantly higher reactive oxygen species (ROS) generation, stronger mitochondrial membrane depolarization, lower dual-specificity phosphatase expression, higher extracellular-signal-regulated kinase (ERK) phosphorylation, lower AKT phosphorylation, and lower expression of the genes encoding the antioxidant enzymes than RA-treated cells. Notably, pretreatment with *N*-acetyl-l-cysteine significantly abolished AgNP-induced neuronal differentiation, but not in that induced by RA. ERK inhibition, but not AKT inhibition, suppresses neurite growth that is induced by AgNP. Taken together, our results uncover the pivotal contribution of ROS in the AgNP-induced neuronal differentiation mechanism, which is different from that of RA. However, the negative consequence of AgNP-induced neurite growth may be high ROS generation and the downregulation of the expression of the genes encoding the antioxidant enzymes, which prompts the future consideration and an in-depth study of the application of AgNP-differentiated cells in neurodegenerative disease therapy.

## 1. Introduction

Neuronal differentiation involves the growth, elongation, and bifurcation of neuronal branches (neurites) out of the neuronal cell body. This process is characterized by various cellular changes, such as morphological changes and the increased expression of neuronal differentiation markers, such as β-tubulin III and microtubule-associated protein 2 (MAP2), which are indispensable for the promotion of neurite growth and maturation [1]. The neuronal differentiation process is essential for the recovery of injured neurons [2].

Several immortalized human cell lines, including neuroblastoma (SH-SY5Y) cells, have been developed for neurobiological studies, avoiding the use of animal (mouse and rat)-derived cell lines, which are not identical to their human counterparts [3]. SH-SY5Y cells possess the capacity for the dopaminergic phenotype [4], and are therefore considered to be a suitable in vitro neurotoxicity model for the study of amyotrophic lateral sclerosis, Parkinson’s disease (PD), and Alzheimer’s disease [5,6]. PD, which is a common neurodegenerative disease, is characterized by the accumulation of α-synuclein-containing abnormal protein aggregates (Lewy bodies) and the loss of dopaminergic neurons from the substantia nigra [7].

All-trans-retinoic acid (RA), brain-derived neurotrophic factor, and phorbol esters are well-known inducers of SH-SY5Y cell differentiation into mature neurons [3,8], and several other materials with this capacity have emerged, including vasoactive intestinal peptide, graphene oxide (GO), silver nanoparticles (AgNP), and GO-AgNP nanocomposites [9,10,11,12]. RA plays important roles in cell proliferation, differentiation, and morphogenesis [13,14]. Moreover, its antioxidant and anti-apoptotic functions have been demonstrated in previous studies [15,16,17].

AgNP have been known for approximately 120 years [18] and are well known for their antibacterial [19], antiviral [20], antimycotic [21], interstitial cystitis reducing [22], anti-inflammatory [23], anti-cancer [24], angiogenesis inducing [25], and wound healing [26] activities. AgNP or other metallic nanoparticles (NPs) are commonplace in the consumer products, such as medical devices, and the occupational exposure to these NPs are associated with health hazards, and so, it is important to delve into the biological impacts of AgNP on human cells [27]. Various recent studies have shed light on the crosstalk between AgNP and cellular differentiation, such as their potency to promote the osteogenic differentiation of stem cells [28].

Here, we synthesized AgNP biologically using *Escherichia coli* (*E. coli*), through the enzymatic reduction of bulk material silver nitrate, which is considered to be an eco-friendly method [29].

Previously, our group and others have reported on the impact of AgNP in the induction of neuronal differentiation [11,12,30]. However, as with most NPs, the mechanism of action remains obscure. In particular, research elucidating the detailed mechanism of AgNP in neuronal differentiation is lacking, and many issues remain to be addressed. Increased understanding of this mechanism will help to evaluate the therapeutic implications of AgNP in neurodegenerative diseases. Accordingly, the main goal of our study is to characterize the mechanism of AgNP induction of SH-SY5Y cell neuronal differentiation in comparison to the commonly used neuronal differentiation-inducing compound, RA. To achieve this goal, we measured the level of reactive oxygen species (ROS) generation, the expression level of the antioxidant genes, mitochondria membrane depolarization, dual-specificity phosphatase (DUSP) expression level, and the activation of AKT and extracellular-signal-regulated kinase (ERK) signaling in AgNP- and RA-differentiated SH-SY5Y cells. Moreover, in an attempt to find out the mechanism that is involved in AgNP-differentiated cells when compared to RA, before the exposure to AgNP or RA, cells were pretreated with various inhibitors, such as *N*-acetyl-l-cysteine (NAC), PD98059, and LY-294002, which target ROS generation, ERK, and AKT signalings, respectively. Our results show a marked difference in the mechanism of the neuronal differentiation induced after the exposure to AgNP or RA and open the door for further studies on NP-induced neuronal differentiation and further clinical applications.

## 2. Results

### 2.1. Influence of Exposure to AgNP or RA on SH-SY5Y Cell Viability and Differentiation

**Figure 1:**
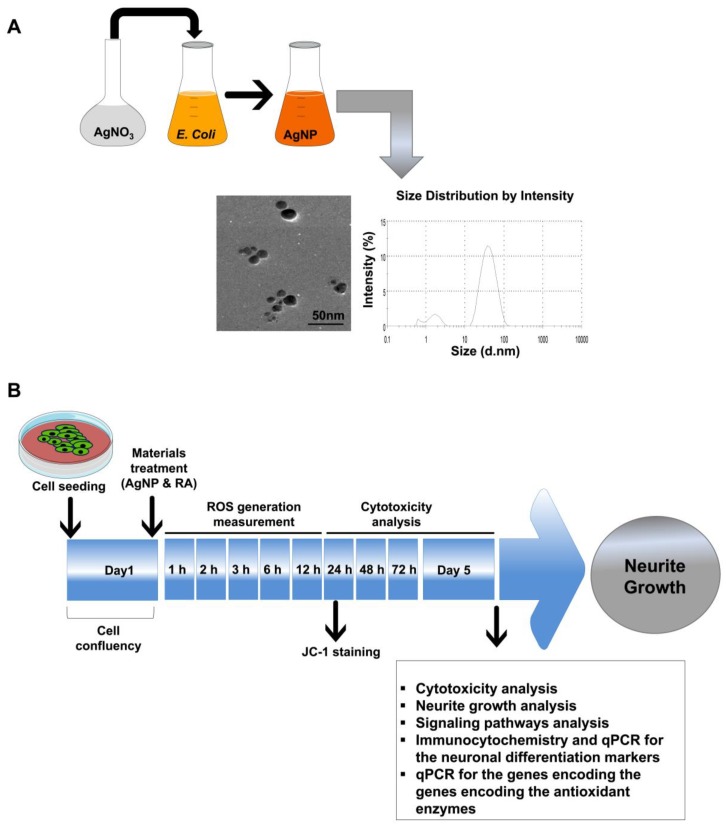
Characterization and experimental scheme for the biologically synthesized silver nanoparticles (AgNP). (**A**) Characterization of the biologically synthesized AgNP. The biological synthesis of AgNP was performed through the reduction of the bulk material silver nitrate (AgNO_3_) by *Escherichia coli* nitrate reductase. Transmission electron microscopy (TEM) imaging of the biologically synthesized AgNP shows a spherical shape with an approximate size of 30 nm. The histogram shows the synthesized AgNP size distribution, as measured by dynamic light scattering (DLS) and presence of small population of the particles between 1 and 3 nm. Scale bar = 50 nm. (**B**) Schematic of the experimental procedures used to compare the neuronal differentiation processes of AgNP- and all-trans-retinoic acid (RA)-exposed neuroblastoma (SH-SY5Y) cells.

**Figure 2:**
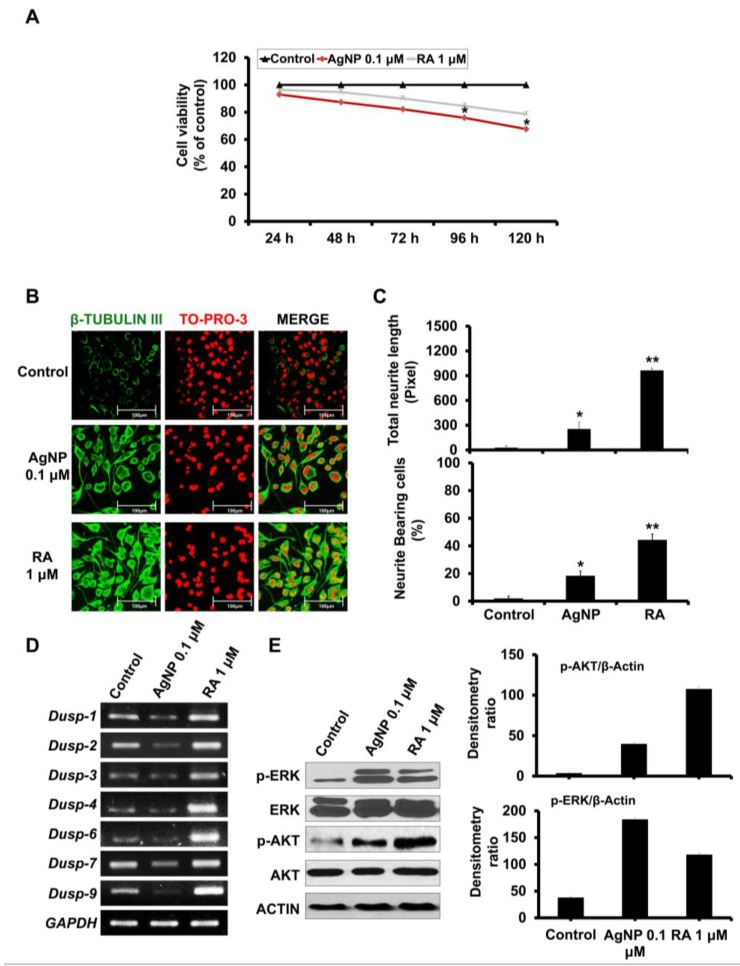
Effects of AgNP and RA on the viability, differentiation, Dual-specificity phosphatase (DUSP expression, and AKT and ERK activation status of SH-SY5Y cells. (**A**) SH-SY5Y cells were incubated with 0.1 μM AgNP or 1 μM RA for 24, 48, 72, 96, and 120 h and viability was analyzed using the EZ-Cytox cell viability kit. SH-SY5Y cells exposed to AgNP for 96 and 72 h showed a significant cytotoxicity. The experiment was performed in triplicate. (**B**) Immunocytochemistry analysis: incubation of SH-SY5Y cells with 0.1 μM AgNP or 1 μM RA for five days. Both RA-exposed and AgNP-exposed cells showed morphological changes (neurite phenotype) and high expression of β-tubulin III. Scale bars, 100 μm. (**C**) Neurite length and the percentage of neurite-bearing cells were measured using the neurite tracing plugin NeuriteTrace in ImageJ. Both AgNP- and RA-exposed cells significantly promoted the neurite length and increased the percentage of neurite-bearing cells. * *p* < 0.05; ** *p* < 0.01. (**D**) Determination of *DUSP1*, *DUSP2*, *DUSP3*, *DUSP4*, *DUSP6*, *DUSP7*, and *DUSP9* expression levels in SH-SY5Y cells after 5 d of incubation with 0.1 μM AgNP or 1 μM RA. *Glyceraldehyde 3-phosphate dehydrogenase (GAPDH)* is a housekeeping gene. *DUSPs* expression level was markedly decreased and increased in AgNP- and RA-treated cells, respectively. (**E**) Western blot analysis was performed to determine the phosphorylation levels of extracellular-signal-regulated kinase (ERK) and AKT in 0.1 μM AgNP- or 1 μM RA-exposed SH-SY5Y cells. Western blot analysis: SH-SY5Y cells treated with 0.1 μM AgNP or 1 mM RA showed high phosphorylation of ERK and AKT signalings. AgNP-exposed cells showed higher phosphorylation of ERK than that shown in RA-exposed cells and higher AKT phosphorylation was detected in RA-exposed cells than that of AgNP-treated cells as depicted in the densitometry analysis (right panel).

We biologically synthesized AgNP with an average size of approximately 30 nm, as characterized by transmission electron microscopy (TEM) and dynamic light scattering (DLS) analyses (Figure 1A). The average zeta potential of our synthesized AgNP was −29.10 mV, which confirms the stability of our AgNP solution. In addition, we detected small population of AgNP between 1 and 3 nm as shown in DLS graph (Figure 1A). After the synthesis and the characterization of the synthesized NPs, the further experiments are summarized in Figure 1B. We first examined changes in SH-SY5Y cell viability after time-dependent exposure (24, 48, 72, 96, and 120 h) to AgNP or RA. Neither AgNP nor RA exposure significantly decreased the viability of SH-SY5Y cells up to 72 h (Figure 2A). In contrast, AgNP-exposed cells showed a significant decrease in the cell viability after 96 and 120 h post treatment. Similarly to RA-exposed cells, AgNP-exposed cells showed morphological changes (neurite phenotype) and the high expression of β-tubulin III, a specific neuronal marker (Figure 2B). However, RA treatment resulted in increased neurite length and a higher percentage of cells bearing neurites when compared to AgNP treatment (Figure 2C).

### 2.2. AgNP and RA Treatment Modulate DUSP Expression Levels and the Activation of Kinase Signaling

*DUSPs* possess a dual role in dephosphorylating phosphor-tyrosine and the phosphor-serine residues and belong to the classical cysteine-related protein phosphatases [31]. The implication of the *DUSPs* in neuronal differentiation and the neuronal diseases is shown in the previous reports [31,32].

We compared the expression levels of seven genes encoding *DUSPs* (*DUSP1*, *DUSP2*, *DUSP3*, *DUSP4*, *DUSP6*, *DUSP7*, and *DUSP9*) in AgNP- and RA-exposed cells. We also examined the phosphorylation levels of AKT and ERK. DUSP expression markedly decreased in AgNP-treated cells, but it increased in RA-treated cells (Figure 2D), while ERK and AKT phosphorylation levels increased in both AgNP- or RA-exposed cells (Figure 2E). However, AgNP-exposed cells showed increased ERK phosphorylation and decreased AKT phosphorylation when compared to cells that were treated with RA, as shown in the densitometry analysis (Figure 2E, right panel).

### 2.3. AgNP and RA Treatment Have Differential Effects on Intracellular ROS Generation, Mitochondrial Membrane Depolarization, and Antioxidant Gene Expression

**Figure 3:**
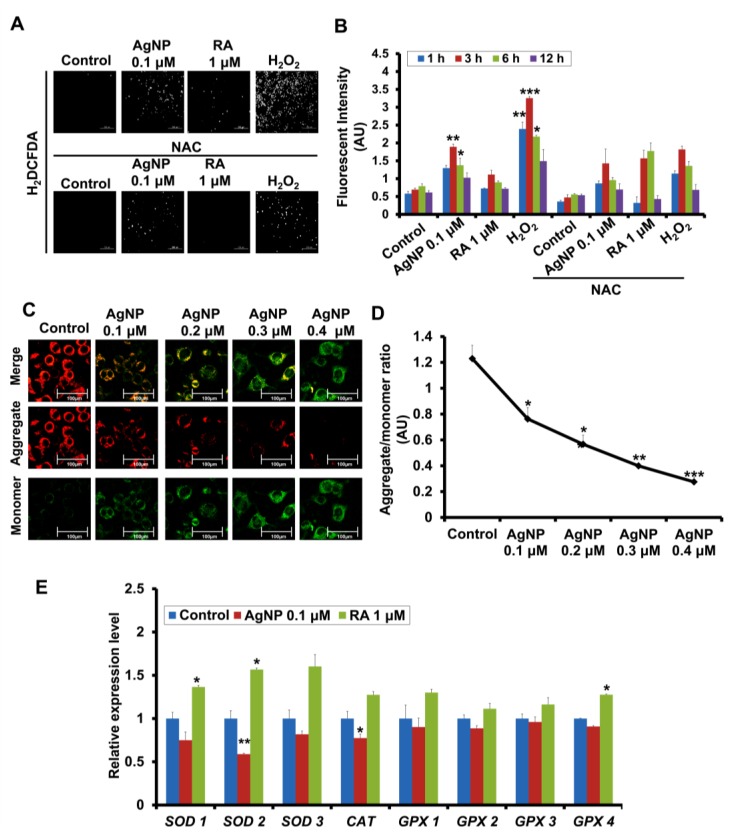
Modulation of reactive oxygen species (ROS) generation, mitochondrial depolarization, and the genes encoding the antioxidant enzymes expression in AgNP- and RA-treated SH-SY5Y cells. (**A**,**B**) ROS generation in SH-SY5Y cells pretreated with NAC (1 mM), and were then treated with 0.1 μM of AgNP or 1 μM of RA or 100 μM of Hydrogen peroxide (H_2_O_2_) was measured by fluorescence microscopy (**A**) and a fluorometric method (**B**). AgNP-exposed cells showed a significant ROS generation, in particular, after 3 h and 6 h of incubation, which is inhibited by NAC pretreatment. * *p* < 0.05; ** *p* < 0.01; *** *p* < 0.001. (**C**) SH-SY5Y cells were incubated with AgNP (0.1, 0.2, 0.3, and 0.4 μM) and the mitochondrial membrane potential (ΔΨm) was measured using JC-1 staining. The qualitative analysis fluorescence intensities of the monomer (green) and an aggregate (red) form was analyzed with the fluorescence confocal microscopy. Scale bars, 100 μm. (**D**) The quantitative analysis of the ratio of aggregate and the monomer was determined using dual-scanning microplate spectrofluorometer. AgNP showed a significant depolarization of the mitochondrial membrane in a dose-dependent manner in SH-SY5Y cells. * *p* < 0.05; ** *p* < 0.01; *** *p* < 0.001. (**E**) Expression of genes encoding the antioxidant enzymes (*SOD1*, *SOD2*, *SOD3*, *CAT*, *GPX1*, *GPX2*, *GPX3*, and *GPX4*) by quantitative real-time PCR. AgNP-treated cells showed a significant suppression of the expression level of *SOD2* and *CAT*. SOD: superoxide dismutase; CAT: catalase; GPX: glutathione peroxidase. * *p* < 0.05; ** *p* < 0.01.

We next analyzed intracellular ROS generation after AgNP and RA treatment. Unlike RA, AgNP-exposed cells exhibited a significant increase in ROS generation after 3 h of exposure (Figure 3A). This was confirmed by spectrophotometric analysis of the fluorescence intensity of ROS signaling (Figure 3B). We confirmed the source of ROS generation by measuring the potency of AgNP mitochondrial membrane depolarization using 5,5′,6,6′-tetrachloro-1,1′,3,3′-tetraethylbenzimidazolcarbocyanine iodide (JC-1), which specifically measures the mitochondrial membrane potential (ΔΨm).

For this purpose, cells were treated with AgNP (0.1, 0.2, 0.3, and 0.4 μM). JC-1 monomer fluorescence emission significantly increased in a dose-dependent manner (Figure 3C), with a low ratio of aggregates/monomers (Figure 3D).

To circumvent the harmful consequences of excessive ROS generation, such as damage to DNA, RNA, proteins, and lipids, various cellular enzymatic defense mechanisms exist to detoxify excess ROS, including enzymatic defense molecules (superoxide dismutase (SOD), catalase (CAT), glutathione peroxidase (GPX), and peroxiredoxin (PRX) and non-enzymatic defense molecules (glutathione, vitamin C, and vitamin E) [33]. The majority of intracellular ROS originates from superoxide (O_2_^−•^), produced by the single electron reduction of O_2_. Copper/zinc SOD (*SOD1*), manganese SOD (*SOD2*), and extracellular SOD (*SOD3*), which are located in the cytosol, mitochondria, and extracellular matrix, respectively [34], play pivotal roles in the conversion of superoxide to hydrogen peroxide (H_2_O_2_) and oxygen. GPX, PRX, and CAT enzymes are responsible for the removal of H_2_O_2_ via its conversion to water and oxygen [35].

Accordingly, we measured the expression levels of the genes that are encoding the antioxidant enzymes *SOD1*, *SOD2*, *SOD3*, *CAT*, *GPX1*, *GPX2*, *GPX3*, and *GPX4* using quantitative real-time polymerase chain reaction (PCR). AgNP- and RA-treated cells showed differential modulation in antioxidant gene expression levels. AgNP-treated cells displayed significantly decreased expression of these enzymes, particularly *SOD2* and *CAT*, but no significant effects on *GPX* expression was detected (Figure 3E). In contrast, RA-exposed cells showed an upregulation of genes encoding the antioxidant enzymes, such as *SODs*, *CAT*, and *GPX4* (Figure 3E).

### 2.4. A ROS Scavenging Agent and ERK and AKT Inhibitors Have Differential Effects on AgNP- and RA-Induced Neuronal Differentiation

The above results indicate the differential modulation of ROS generation and ERK and AKT phosphorylation in AgNP- and RA-exposed cells. Accordingly, we next characterized the importance of ROS generation and the phosphorylation of ERK and AKT on AgNP- or RA-induced neuronal differentiation via pretreatment with inhibitors that were targeting these elements.

**Figure 4:**
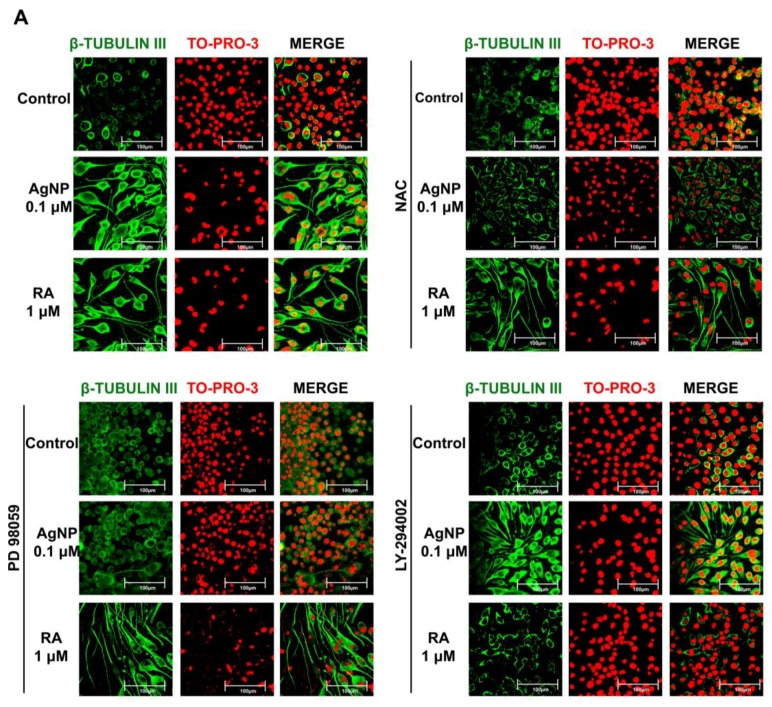

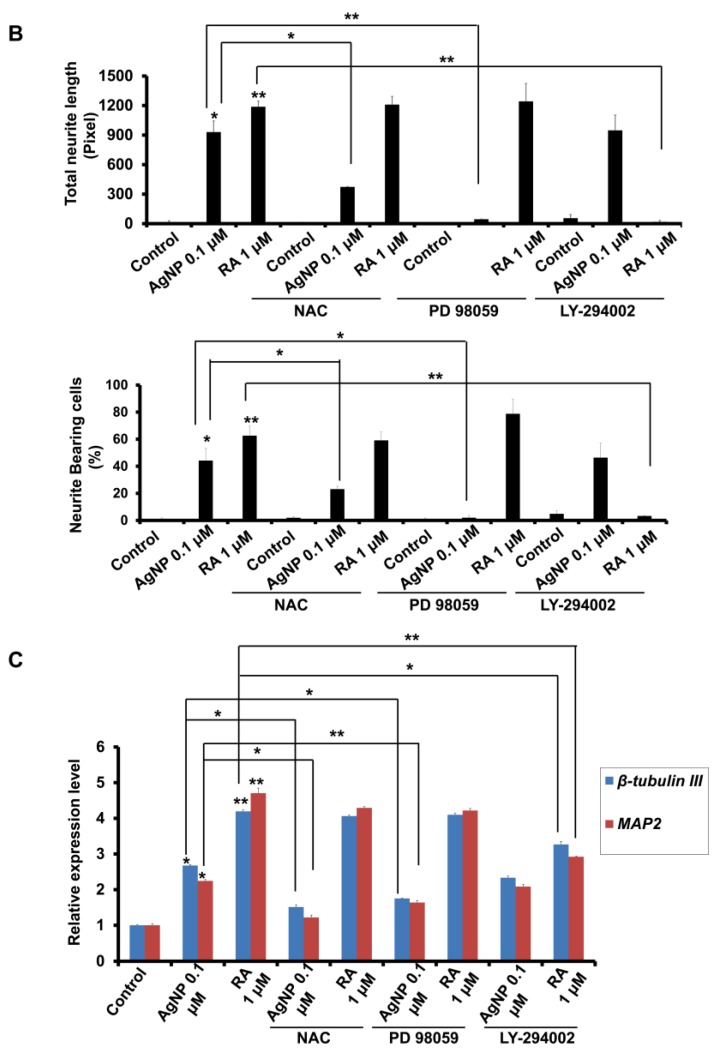
Roles of inhibitors of ROS generation and ERK/AKT signaling in the modulation of AgNP- and RA-induced neurite growth. (**A**) Immunocytochemistry analysis of the expression level of the neuronal differentiation marker β-tubulin III (green florescence) of SH-SY5Y cells pretreated with the antioxidant NAC (1 mM), the ERK inhibitor PD98059 (10 μM), or the AKT inhibitor LY-294002 (10 μM) for 1 h, and then exposed to 0.1 μM AgNP or 1 μM RA for five days. Scale bars, 100 μm. (**B**) Neurite length and the percentage of neurite-bearing cells were measured using the neurite tracing plugin NeuriteTrace in ImageJ. The pretreatment of NAC and PD98059 significantly abolished AgNP-induced neurite growth and high neurite-bearing cells percentage, but LY-294002 pretreatment markedly suppressed the RA-induced neurite growth. * *p* < 0.05; ** *p* < 0.01. (**C**) The expression of the neuronal differentiation-specific markers *microtubule-associated protein 2 (MAP2)* and *β-tubulin III* was measured by quantitative real-time PCR in cells that were pretreated and AgNP- or RA-exposed, as above. The expression level of the neuronal differentiation markers in AgNP- and RA-treated cells after the pretreatment of the aforementioned inhibitors confirmed the immunocytochemistry results. * *p* < 0.05; ** *p* < 0.01.

First, we examined the contribution of ROS to AgNP- and RA-induced neuronal differentiation by pretreating the cells with the ROS scavenging agent NAC. Immunofluorescence analysis revealed that NAC pretreatment led to significant reductions in neurite growth and β-tubulin III expression in AgNP-differentiated cells (Figure 4A). In contrast, NAC pretreatment did not have any significant effect on RA-induced neuronal differentiation (Figure 4A).

To determine the importance of ERK and AKT signaling in AgNP- and RA-differentiated cells, we pretreated cells with ERK (PD98059) and AKT (LY-294002) pathway inhibitors. PD98059 pretreatment markedly suppressed neurite growth and β-tubulin III expression in AgNP-differentiated cells, while pretreatment with LY-294002 had no effect (Figure 4A). Conversely, LY-294002 pretreatment, but not PD98059 pretreatment, decreased the neurite growth and β-tubulin III expression in RA-treated SH-SY5Y cells (Figure 4A). The analysis of neurite length and the percentage of neurite-bearing cells were carried out using the neurite tracing plugin NeuriteTrace in ImageJ (Figure 4B). Moreover, MAP2 and β-tubulin III expression levels were significantly decreased upon PD98059 pretreatment in AgNP-differentiated cells only, which was confirmed by quantitative real time PCR (Figure 4C). In RA-differentiated cells, LY-294002, but not PD98059 pretreatment, suppressed the expression of neuronal differentiation markers (Figure 4C).

**Figure 5:**
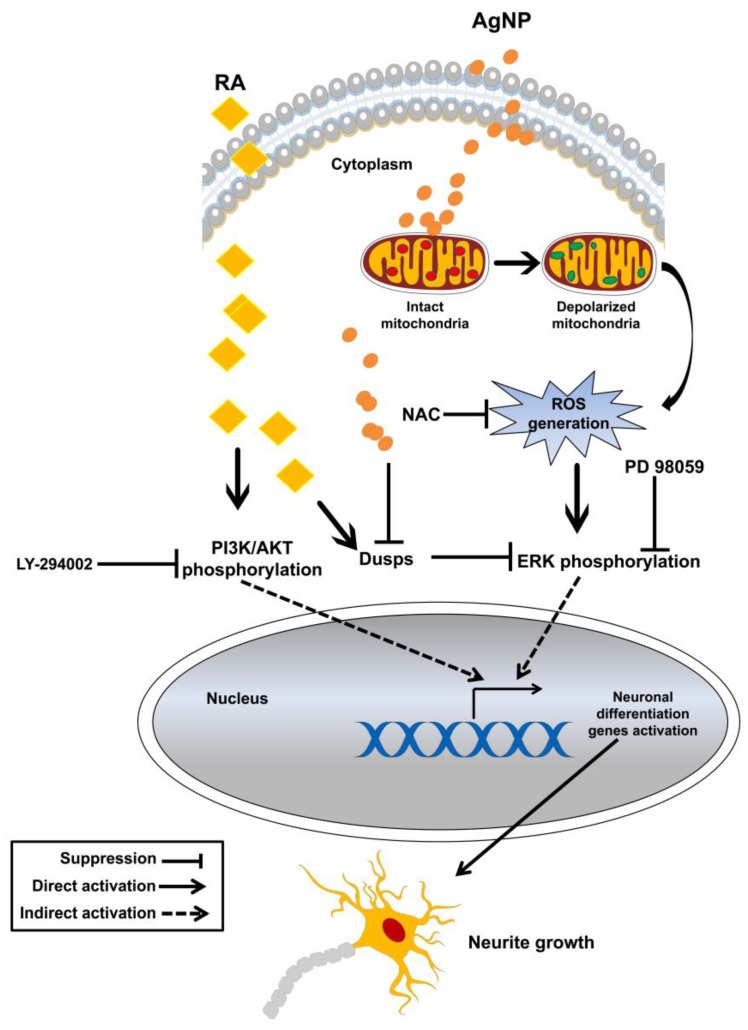
Hypothetical model of the mechanisms of AgNP- and RA-induced neuronal differentiation of SH-SY5Y cells.

Taken together, the results suggest that AgNP- and RA-differentiated cells displayed differential mechanisms of neuronal differentiation induction; in particular, ROS and ERK signaling are essential for AgNP-induced neurite growth and AKT signaling is mainly involved in RA-related neurite growth (Figure 5).

## 3. Discussion

NPs possess high functionality when compared to the original bulk particle (inert material), which is attributed to their small size and large surface area. There is a plethora of scientific reports that examine the link between NPs and the promotion of the neuronal differentiation, as well as their possible application in nerve injury repair, which is a challenging problem in the neuroscience [36,37,38]. For instance, iron oxide NP treatment led to increased neurite growth and an increase in the number of neurite-bearing PC12 cells [39]. Gold nanorod-exposed NG108-15 cells that were cultured on dishes coated with either poly (4-styrenesulfonic acid) or SiO_2_ show 20–25% increase in neurite outgrowth when compared to cells not exposed to gold nanorods [40].

Several plausible mechanisms regarding the potency of NPs to induce neuronal differentiation and increase neurite growth have been proposed, such as transcription factor activation [40], changes in the expression of cytoskeleton-related genes, and changes in growth hormone receptor signaling [39].

AgNPs are well-known for their excellent antibacterial activity, which is attributed to the release of silver ions (Ag^+^), slow oxidation, and binding and penetration of the cell membrane, leading to bacterial cell damage and death [41,42]. Despite their wide range of biological functions and clinical applications, higher concentrations of AgNP have harmful and deleterious health effects [43]. However, the toxic action of AgNP depends on the particle size, surface area, concentration, and the exposure time [44,45,46]. 

In our study, AgNPs were biologically synthesized by reducing AgNO_3_ to Ag with an average size of 30 nm (Figure 1A). We previously showed the potential of these biologically synthesized AgNP in stimulating neurite outgrowth via the upregulation of specific neuronal differentiation markers and increased activation of ERK and AKT signaling. We also demonstrated their potential to generate ROS [11].

A wide range of microbes, such as bacteria, fungi, and algae represents a factories for reduction and the ultimate green synthesis of the NPs [47]. A recent interesting report biologically synthesized gold NPs using glycolipids that purified from *lactobacillus casei* and produced small size NPs [48]. The biological synthesis of the NPs avoids using toxic chemicals and produces a stable NPs, which is attributed to the biomolecules in the culture supernatant [49,50].

Consistent with our findings, AgNP have been shown to play an important function in the initiation and elongation phases of SH-SY5Y cells, with no cytotoxicity [12,30,51].

Several studies show the impact of AgNP on differentiation of the mesenchymal stem cells [52,53,54,55,56] and the proliferation and toxicity of stem cell [57,58,59]. Of the note, there are various reports showing the molecular functions of the biologically synthesized AgNP. For instance, a biologically synthesized AgNP using *E. coli* efficiently induced the cytotoxicity of F9 teratocarcinoma stem cells in a dose-increment manner, which attributed to high ROS generation, mitochondrial dysfunction, and high production of the lactate dehydrogenase [60]. On the other hand, F9 teratocarcinoma stem cells that were exposed to low concentration of AgNP differentiated into a neuronal lineage, which was evidenced by the high expression of the neuronal specific markers [60]. Another interesting study proved the potential of the biologically synthesized AgNP in the induction of cytotoxicity in human lung epithelial adenocarcinoma cell line in a dose-dependent manner, which is mediated by induction of ROS generation, depolarization of the mitochondrial membrane, and the production of the lactate dehydrogenase [61].

AgNP synthesized with *Bacillus licheniformis* inhibited the angiogenesis when treated with 500 nM that shown in the inhibition to the growth and the migration of the bovine retinal endothelial cells with and without treatment of the vascular endothelial growth factor [62]. In addition, the anti-cancer capacity of the biologically synthesized AgNP using bacteria via various mechanisms is shown in several literatures [63,64,65]. The cellular responses and the biological functions of AgNP in a wide range of cells lines, including, normal, cancer, and stem cells are summarized and reviewed elsewhere [45]. As data demonstrating the relevance of AgNP to neurite growth continue to accrue, it will become essential to determine the factors, which are relevant to AgNP function in promotion of the neuronal growth. To achieve this purpose, we examined the mechanism of AgNP-induced neurite growth when compared to RA-induced differentiation.

AgNP- or RA-exposed SH-SY5Y cells did not show significant suppression of cell viability. We confirmed the potential of AgNP to induce neurite growth by immunofluorescence staining of the neuronal differentiation marker β-tubulin III. Of note, the effect of AgNP on ROS generation and cytotoxicity is a concentration-dependent concept. In this regard, our previous study showed that, as the exposure dose of AgNP increased, the cell toxicity significantly increased [11]. Our current results revealed that the cytotoxicity was significantly increased as the time of exposure increased, in particular, with 96 and 120 h of exposure (Figure 2A). In addition, we detected a significant depolarization of the mitochondrial membrane in a dose-increment manner (Figure 3C,D).

The dephosphorylation of phosphoserine, phosphothreonine, and phosphotyrosine residues on kinases is mainly performed by the *DUSPs*, which are a subclass of the protein tyrosine phosphatase superfamily [66]. AgNP-treated SH-SY5Y cells displayed decreased DUSP expression, while RA-exposed cells displayed increased expression. The upregulation of DUSP by RA treatment is shown previously [32], and the modulation of DUSP by NPs is reported [67]. Both RA and AgNP treatments resulted in the activation of AKT and ERK signaling; however, AgNP caused higher ERK phosphorylation and lower AKT phosphorylation than RA. The crosslink between AgNP exposure and ERK activation is shown in the previous reports [25,68,69]. The AKT pathway is an important component of RA signaling [70,71], which is consistent with our results.

Recently, rather than focusing on their deleterious effects, reports on the physiological roles of ROS in cellular function and differentiation have increased. ROS generation can modulate osteogenesis [72], neuronal differentiation [73], and neurogenesis [74], and its optimal concentration is important for the stimulation of crucial signaling pathways, and consequently, the regulation of various cellular functions, such as proliferation, differentiation, and cell death [75,76]. ROS generation was significantly increased upon AgNP treatment but not RA treatment. Moreover, JC-1 staining suggested an interaction between AgNP and the mitochondrial membrane, leading to its depolarization. Additionally, a significant suppression of the expression of antioxidant genes, such as SOD and CAT, was detected in AgNP-exposed cells, while they were significantly upregulated in RA-treated cells. The link between RA and the upregulation of *SOD2* has also been reported previously [77,78]. Collectively, the results suggest that the mitochondria may be involved in AgNP-induced ROS generation, as consistent with previous reports showing the role of the mitochondria in AgNP-induced ROS generation [79,80].

To reveal the role of ROS signaling in AgNP-induced neuronal differentiation, treatment with the ROS scavenger NAC was performed before AgNP exposure. The neurite growth enhancement that was observed with AgNP was abolished by NAC pretreatment, which did not affect RA-differentiated cells. A link between ROS generation and ERK activation has been previously reported [81,82,83], implicating ERK signaling in the promotion of neuronal differentiation and nervous system development [83,84].

In conclusion, AgNPs interact with the mitochondrial membrane, leading to mitochondrial membrane polarization, and resulting in intracellular ROS generation. ROS generation may increase ERK phosphorylation and suppress DUSP expression, thus enhancing the expression of neuronal differentiation genes and resulting in neurite growth. The signaling pathway that is involved in the AgNP-mediated neuronal differentiation of SH-SY5Y cells differs from that of RA-mediated differentiation, in which activation of AKT signaling and high DUSP expression ultimately upregulates the expression of neuronal differentiation genes (Figure 5). Our study also highlights the impact of ROS on AgNP-induced neurite growth and provides a model that can be applied in future studies to explore the biological functions of various NPs possessing ROS generation potential, such as silicate, gold, and iron oxide NPs, in neuronal differentiation, and their possible therapeutic applications in neurodegenerative diseases. There is a broad spectrum of microorganisms that needs further studies for characterization of their roles in the biological synthesis of NPs and their mechanisms need to be revealed as well. Also, the larger scale application of bacterial-based NPs synthesis needs further consideration. Both AgNP and RA are in common in the promotion of the neurite growth. However, increased cytotoxicity, enhanced oxidative stress, and decreased expression of genes encoding the antioxidant enzymes after AgNP treatment could be a negative effect of AgNP-induced differentiation, and this will open the door for novel in-depth studies on the suitability of the nanomaterials in the treatment of neurodegenerative disorders in the future.

## 4. Materials and Methods

### 4.1. Materials

Fetal bovine serum (FBS) and high-glucose Dulbecco’s modified Eagle medium (DMEM) were purchased from Hyclone (Logan, UT, USA) for SH-SY5Y cell culture. Penicillin (50 U/mL) and streptomycin (50 μg/mL) were obtained from Invitrogen (Carlsbad, CA, USA). RA, NAC, PD98059, LY-294002, and epoxomicin were purchased from Sigma-Aldrich (St. Louis, MO, USA).

### 4.2. AgNP Synthesis and Characterization

AgNPs were biologically synthesized, as previously described [11,85]. In brief, the culture of *E. coli* was carried out using Luria-Bertani medium (without sodium chloride), which was incubated for 21 h at 37 °C at 120 rpm on the shaker. Then, the culture supernatant, which is used for AgNP synthesis, was prepared via the centrifugation at 10,000 rpm. For AgNP synthesis, 1 mM AgNO_3_ solution was added to the culture supernatant and incubated for 24 h. The synthesized AgNP was characterized by Biochrom WPA Biowave II UV-vis spectroscopy (Biochrom, Cambridge, UK). TEM (JEM-1200EX; EM Lab Services, Topeka, KS, USA) to determine the shape and size of the synthesized AgNP and the particle size was analyzed by DLS on a Zetasizer Nano ZS90 (Malvern Instruments, Worcestershire, UK), as previously described [11]. The zeta potential of AgNP was measured with a zeta potential analyzer (ELSZ-1000, Otsuka Electronics Co., Ltd., Osaka, Japan).

### 4.3. SH-SY5Y Cell Culture and Treatment with AgNP and RA

Human neuroblastoma SH-SY5Y cells were cultured in DMEM supplemented with 10% FBS, penicillin (50 U/mL), and streptomycin (50 μg/mL), and were maintained at 37 °C with 5% CO_2_. The AgNP/RA treatment protocol is illustrated in Figure 1B. Prior to the neuronal differentiation induction with 0.1 μM AgNP or 1 μM RA, the growth medium was exchanged with medium containing 1% FBS. AgNP and RA were diluted to the indicated concentrations using this medium and were incubated with the cells for five days.

### 4.4. Cytotoxicity Assays

Cells were treated with AgNP or RA at the indicated concentrations for various time points in triplicate wells and were analyzed for cytotoxic effects using the EZ-Cytox cell viability kit (Daeil Lab Service, Seoul, Korea). SH-SY5Y cells were seeded onto 96-well plates at 1 × 10^4^ cells/well, incubated overnight to reach confluence, and then treated with media containing 0.1 μM AgNP or 1 μM RA for 24, 48, 72, 96, and 120 h. Afterward, the treated medium was replaced with fresh medium containing 10% EZ-Cytox and was incubated at 37 °C for 3 h with 5% CO_2_ in the dark. To quantify the cell viability, the optical densities of the treated wells were measured at 480 nm using a Bio-RAD x-Mark^TM^ spectrophotometer (Bio-Rad Laboratories, Hercules, CA, USA). AgNP- and RA-treated cells were compared with mock-treated cells, which were arbitrarily set to 100% viability.

### 4.5. Immunofluorescence Staining

For immunofluorescence, glass slides (12/12 mm, Superior-Marienfeld, Lauda-Königshofen, Germany) were rinsed in 80% ethanol for 15 min, washed with distilled water, dried, and stored in a dry place until use. The glass slides were placed in the wells of 12-well plates, and SH-SY5Y cells (4 × 10^4^ cells/well) were seeded onto the prepared glass slides and incubated overnight. After five days of differentiation, the cells were prepared for immunofluorescence, as described previously [11]. Normal goat serum (10%) was used as a blocking solution. After incubation with a primary monoclonal antibody against β-tubulin III (Sigma Aldrich, St. Louis, MO, USA), the cells were incubated with secondary antibody (Dylight 549-conjugated goat anti-mouse antibody, Jackson ImmunoResearch Labs, West Grove, PA, USA). TO-PRO-3 (Molecular Probes, Eugene, OR, USA) was used for nuclear staining, and images were captured using a Leica laser scanning confocal microscope.

### 4.6. Neurite Growth Analysis

After immunofluorescence staining for neuronal differentiation markers, the neurite length in AgNP- or RA-exposed cells was calibrated using the neurite tracing plugin NeuriteTrace in ImageJ software (Version 1.50i, National Institutes of Health, Bethesda, MD, USA) [86]. To calculate the percentage of neurite bearing cells, we divided the number of cells that displayed visually distinguishable neurites by the total number of cells in the field, and then multiplied by 100 to obtain the percentage. Neurite-bearing cells were selected when they possessed at least one neurite growth that was longer than the cell body.

### 4.7. Intracellular ROS Measurements and Quantification of Antioxidant Gene Expression

To monitor intracellular ROS levels, we used a fluorescent probe, 2′,7′-dichlorofluorescin diacetate (H_2_DCFDA; Molecular Probes, Eugene, OR, USA), which is cell-permeable and is broadly used to monitor the levels of intracellular ROS, such as hydrogen peroxide (H_2_O_2_) [87] and various reactive intermediates [88]. Before exposure to AgNP or RA or 100 μM H_2_O_2_ (Sigma Aldrich, St. Louis, MO, USA) for 1, 3, 6, and 12 h, SH-SY5Y cells were pretreated with NAC (1 mM) for 1 h and then incubated with 10 μM H_2_DCFDA at 37 °C for 30 min in the dark. Afterward, the cells were washed twice, covered with PBS, and the fluorescence intensity was analyzed using a Spectra MAX Gemini EM (Molecular Devices, San Jose, CA, USA) dual-scanning microplate spectrofluorometer with excitation and emission at 490 and 530 nm, respectively. Fluorescent images were captured on a Nikon Eclipse TE2000-U fluorescent inverted microscope (Nikon, Tokyo, Japan). In addition, we measured the expression of genes that were encoding the antioxidant enzymes, including *SOD1*, *SOD2*, *SOD3*, *CAT*, *GPX1*, *GPX2*, *GPX3*, and *GPX4* after AgNP and RA treatment using quantitative real-time PCR analysis.

### 4.8. RNA Extraction, cDNA Synthesis, and mRNA Expression Analysis

Total RNA was isolated from cells using the Easy-Blue total RNA extraction kit (iNtRON Biotechnology, Seongnam, Korea), according to the manufacturer’s instructions and quantified on a NanoDrop (ND1000) spectrophotometer (Nanodrop Technologies Inc., Wilmington, DE, USA). cDNA was synthesized from 2 μg total RNA using MMLV reverse transcriptase (Promega, Madison, WI, USA) and oligo (dT). PCR amplification was performed using 2× PCR Master Mix Solution (Elpis Biotech, Daejeon, Korea), and products were analyzed on 1–2% agarose gels. For quantitative real-time PCR, the quantification of expression changes was performed using SYBR Green master mix (Elpis Biotech, Daejeon, Korea) and an Applied Biosystems 7500 real-time PCR system. The expression level of target genes was normalized to the housekeeping gene *glyceraldehyde 3-phosphate dehydrogenase* (*GAPDH*), and the calculation of the relative expression was performed using the comparative Ct or ΔΔCt method. All primer sequences used are listed in Table 1.

### 4.9. ΔΨm Analysis Using JC-1

The aim of this experiment is to measure the alteration in ΔΨm after the exposure to various concentrations of AgNP. Changes in ΔΨm were investigated using the lipophilic cationic carbocyanine dye JC-1 (Molecular Probes, T-3168), which is a ratiometric tool for measuring mitochondrial polarization [89]. Upon excitation at 490 nm, J-aggregates emit red fluorescence at 590 nm in intact mitochondria and J-monomers emit a green fluorescence at 540 nm in the depolarized mitochondria.

For the qualitative fluorescence analysis, SH-SY5Y cells were seeded onto the prepared glass slides, which were placed in the wells of 12-well plates and were incubated overnight. The preparation of the glass slides was explained in detail in the immunofluorescence staining section. After treating SH-SY5Y cells with 0.1, 0.2, 0.3, and 0.4 µM AgNP for 24 h, JC-1 was added to serum-free medium to a final concentration of 10 μM and then incubated at 37 °C for 15 min. The images of the fluorescent signals were captured with Leica laser scanning confocal microscope (Leica Microsystems, Wetzlar, Germany).

For the quantitative measurement of the fluorescent intensity, SH-SY5Y cells were seeded onto 96-well plate and were subjected to AgNP treatments and JC-1 staining, as described before. Afterward, the fluorescent intensities were measured with a Spectra MAX Gemini EM dual-scanning microplate spectrofluorometer [90]. The ratio of the fluorescent intensities between the monomers (red) and the aggregates (green) is an indicator of ΔΨm [91]. The low ratio of red/green florescent intensities indicate the mitochondrial depolarization 

### 4.10. Western Blot Analysis

Total protein was extracted from AgNP- and RA-exposed cells, as previously described [11], and the protein concentration of the supernatant was quantified using Bradford protein assay reagent (Bio-Rad Laboratories). Lysates (40 µg protein) were resolved by sodium dodecyl sulfate-polyacrylamide gel electrophoresis and were then transferred onto nitrocellulose membranes. The membranes were then blocked using 5% (*w*/*v*) skimmed milk dissolved in Tris-buffered saline (TBS) with 1% (*v*/*v*) Tween-20 (TBST) for 1 h; incubated overnight with anti-phosphorylated AKT, anti-AKT, anti-phosphorylated ERK1/2, anti-β-Actin (all Santa Cruz Biotechnology, Dallas, TX, USA), and anti-ERK1/2 (Cell Signaling Technology, Beverly, MA, USA) primary antibodies, and then incubated with horseradish peroxidase-conjugated secondary antibodies (Santa Cruz Biotechnology) for 2 h. All of the antibodies were diluted at the manufacturers’ recommended concentrations in 5% (*w*/*v*) dry milk dissolved in PBST. Protein signals were detected on X-ray films using an enhanced chemiluminescence kit (Amersham Biosciences, Piscataway, NJ, USA). The densitometry analysis was carried out using ImageJ software and the graphic data represent the ratio between the intensity of the proteins and the intensity of the housekeeping protein, β-Actin.

### 4.11. Statistical Analyses

All of the experiments were performed in three independent times. Excel program 2010 (Microsoft, Redmond, WA, USA) was used to calculate the mean ± standard deviation of the obtained data. Data are presented as the mean ± standard deviation (SD). All of the statistical analyses were carried out with GraphPad InStat V. 3.0 software (GraphPad Software, San Diego, CA, USA) using one-way analysis of variance (ANOVA) or two-tailed Student’s *t*-test. The data were considered significant at *p* < 0.05.

## Figures and Tables

**Table 1:** Primer sequences for neuronal differentiation markers, *DUSPs* genes, and oxidative-related genes.

	Genes	Forward Primer	Reverse Primer
Neuronal markers	*MAP2*	GCTCTCTGAAGAACATCCGC	GGGCTTTAGCATGCTCTCTG
	*β-tubulin III*	TCTCACAAGTACGTGCCTCG	CTCCGTGTAGTGACCCTTGG
*DUSPs*-related genes	*Dusp-1*	ACCACAAGGCAGACATCAG	AAGGTAAGCAAGGCAGATGG
	*Dusp-2*	CAGCTGCTGCAGTTTGAGAC	AGCTGATTTCTGCCAGAGGA
	*Dusp-3*	GATCTCAACGACCTGCTCTC	ATGGGTGATGCCTAGTTTCTG
	*Dusp-4*	ACGGCTCTGTTGAATGTCTC	CAGTCCTTCACGGCATCG
	*Dusp-6*	ACAAGCAAATCCCCATCTCG	CAGCCAAGCAATGTACCAAG
	*Dusp-7*	AACCTACCCAACGCCTTC	CACCAGGACACCACACTTC
	*Dusp-9*	GAGGCTTCAGCAGATTCCAG	ATTGAGGATGTAGCGGATGC
Oxidative-related genes	*SOD1*	GGTGGGCCAAAGGATGAAGAG	CCACAAGCCAAACGACTTCC
	*SOD2*	GCTCCGGTTTTGGGGTATCTG	GCGTTGATGTGAGGTTCCAG
	*SOD3*	ATGCGTGCGCTACTGTGTTC	CTCCGCCGAGTCAGAGTTG
	*CAT*	CTCCGCCGAGTCAGAGTTG	CCTTTGCCTTGGAGTATTTGGTA
	*GPX1*	CAGTCGGTGTATGCCTTCTCG	GAGGGACGCCACATTCTCG
	*GPX2*	GGTAGATTTCAATACGTTCCGGG	TGACAGTTCTCCTGATGTCCAAA
	*GPX3*	AGAGCCGGGGACAAGAGAA	ATTTGCCAGCATACTGCTTGA
	*GPX4*	GAGGCAAGACCGAAGTAAACTAC	CCGAACTGGTTACACGGGAA
Housekeeping gene	*GAPDH*	AATCCCATCACCATCTTCCAG	AAATGAGCCCCAGCCTTC

## Abbreviations

AgNP	silver nanoparticle
RA	all-trans-retinoic acid
ROS	reactive oxygen species
DUSP	dual-specificity phosphatase
NAC	*N*-acetyl-l-cysteine
ERK	extracellular-signal-regulated kinase
AKT	AK mouse strain transforming
PD	Parkinson’s disease
SH-SY5Y	human neuroblastoma cell line
GO	graphene oxide
TEM	transmission electron microscopy
DLS	dynamic light scattering
SOD	superoxide dismutase
CAT	catalase
GPX	glutathione peroxidase
PRX	Peroxiredoxin
MAP2	Microtubule-associated protein 2
H_2_O_2_	hydrogen peroxide
PD98059	ERK inhibitor
LY-294002	AKT inhibitor
H_2_DCFDA	2′,7′-dichlorofluorescin diacetate
ΔΨm	mitochondrial membrane potential
DMEM	Dulbecco’s modified Eagle’s medium
FBS	fetal bovine serum
PBS	phosphate-buffered saline
TBS	Tris-buffered saline
JC-1	5,5′,6,6′-tetrachloro-1,1′,3,3′-tetraethylbenzimidazolylcarbocyanine iodide
